# Supplementary material for: Astrocytic Cholesterol Fine-Tunes the Balance of Different Modes of Synaptic Exo- and Endocytosis
Source: bioRxiv. 2026 Feb 7:2025.12.28.696787. Preprint. [Version 2] doi: 10.64898/2025.12.28.696787 (PMC12884330; doi:10.64898/2025.12.28.696787)
Supplement: 1 [file NIHPP2025.12.28.696787V2-supplement-1.pdf]

(which was not certified by peer review) is the author/funder, who has granted bioRxiv a license to display the preprint in perpetuity. It is m
available under aCC-BY 4.0 International license.

Supplementary Figure 1

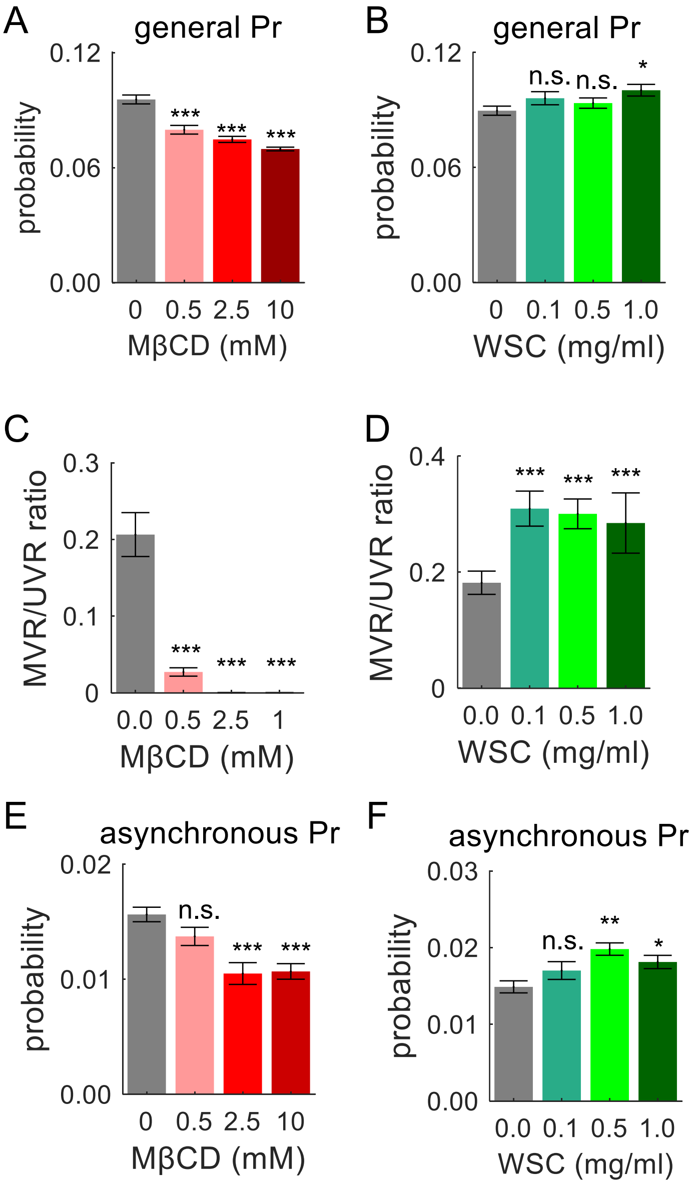

(which was not certified by peer review) is the author/funder, who has granted bioRxiv a license to display the preprint in perpetuity. It is m
available under aCC-BY 4.0 International license.

Supplementary Figure 2

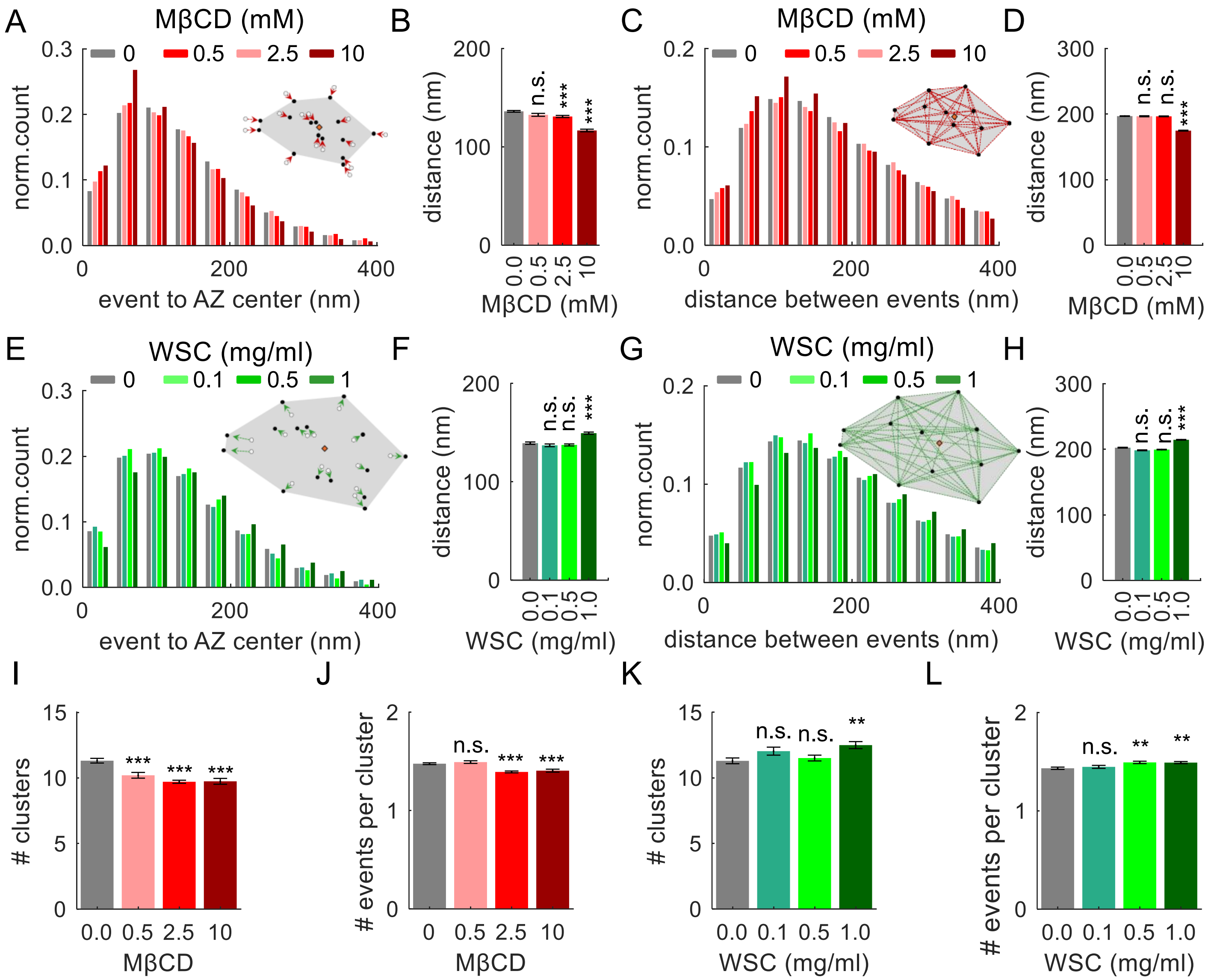

(which was not certified by peer review) is the author/funder, who has granted bioRxiv a license to display the preprint in perpetuity. It is m
available under aCC-BY 4.0 International license.

Supplementary Figure 3

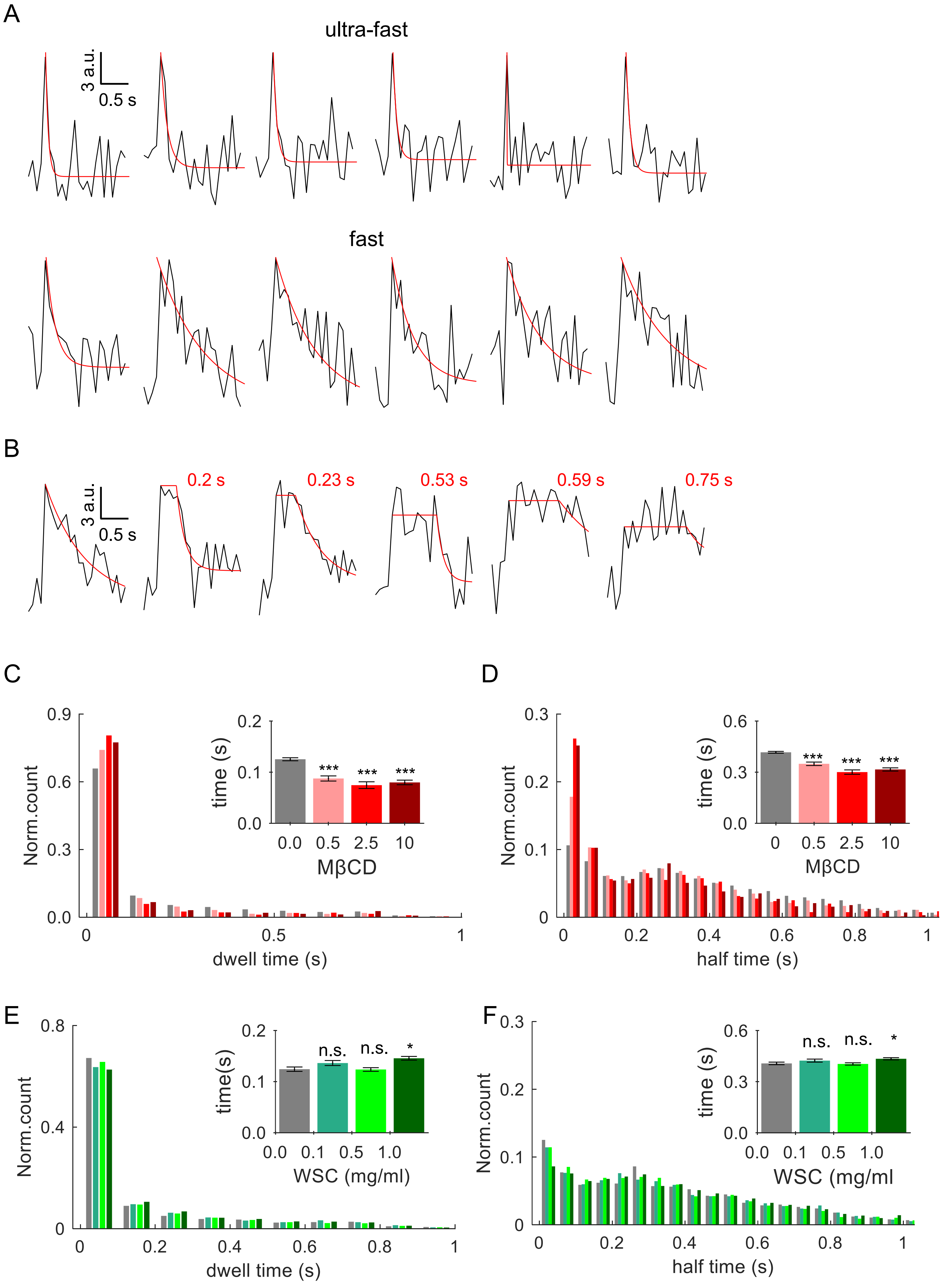

(which was not certified by peer review) is the author/funder, who has granted bioRxiv a license to display the preprint in perpetuity. It is m
available under aCC-BY 4.0 International license.

Supplementary Figure 4

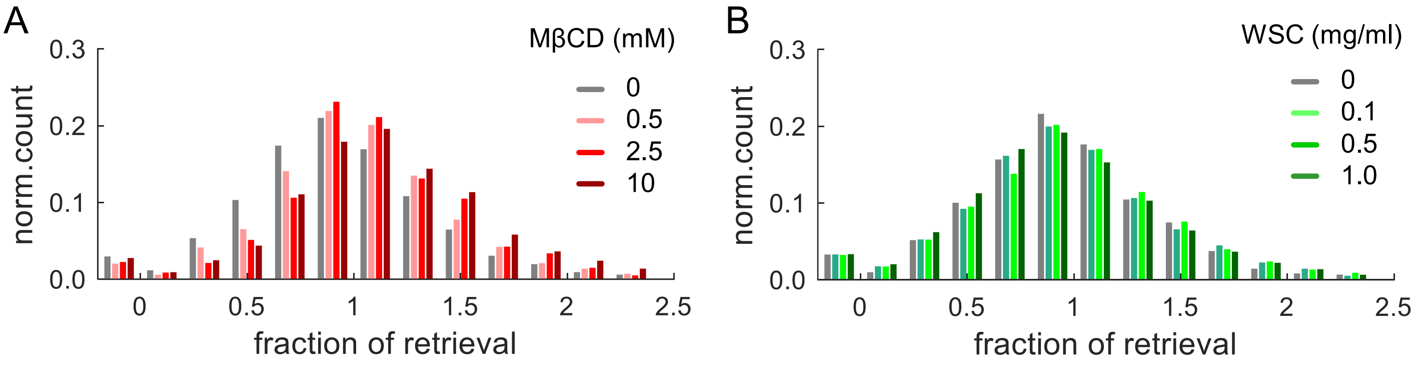

(which was not certified by peer review) is the author/funder, who has granted bioRxiv a license to display the preprint in perpetuity. It is m
available under aCC-BY 4.0 International license.

Supplementary Figure 5

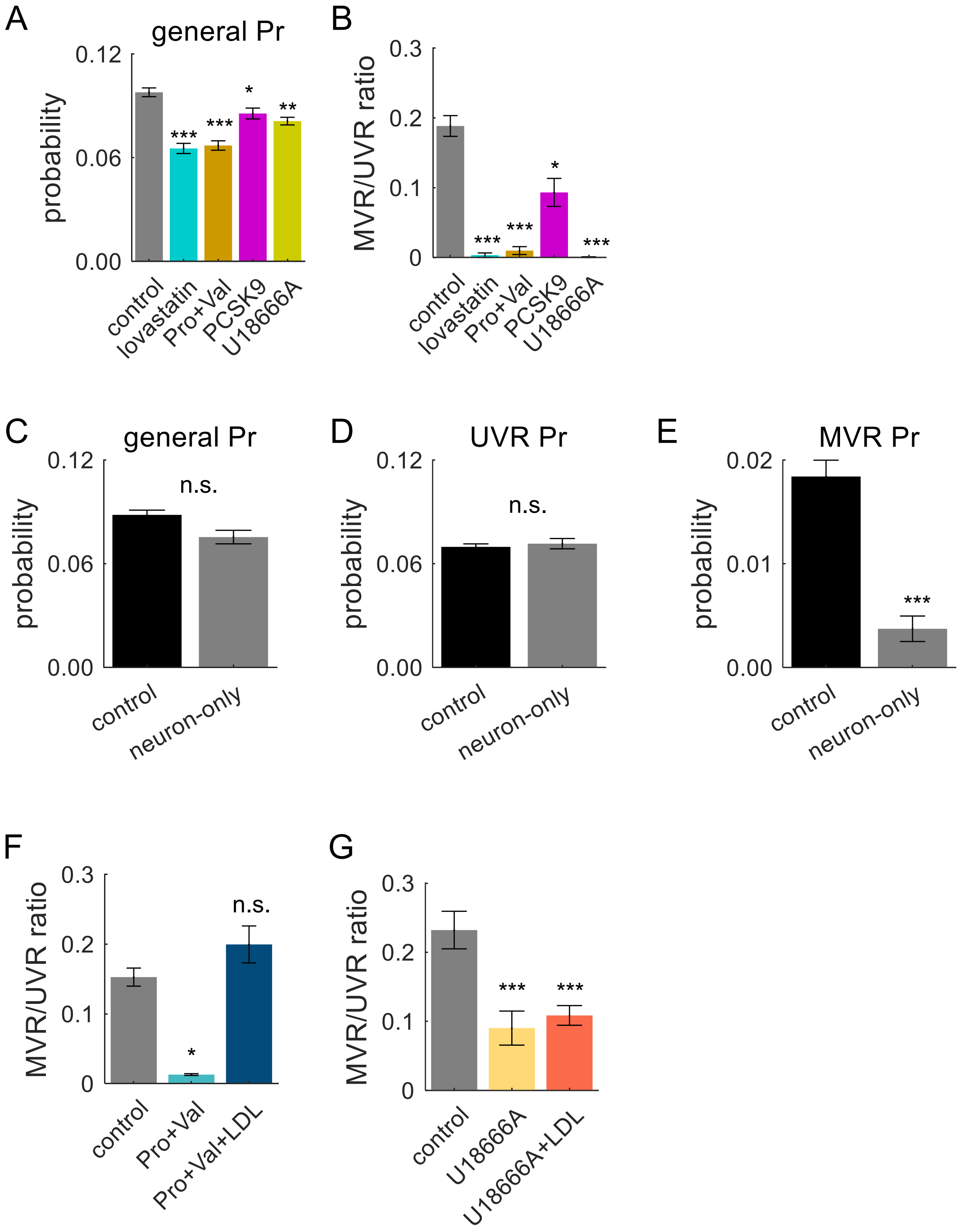

(which was not certified by peer review) is the author/funder, who has granted bioRxiv a license to display the preprint in perpetuity. It is m
available under aCC-BY 4.0 International license.

Supplementary Figure 6

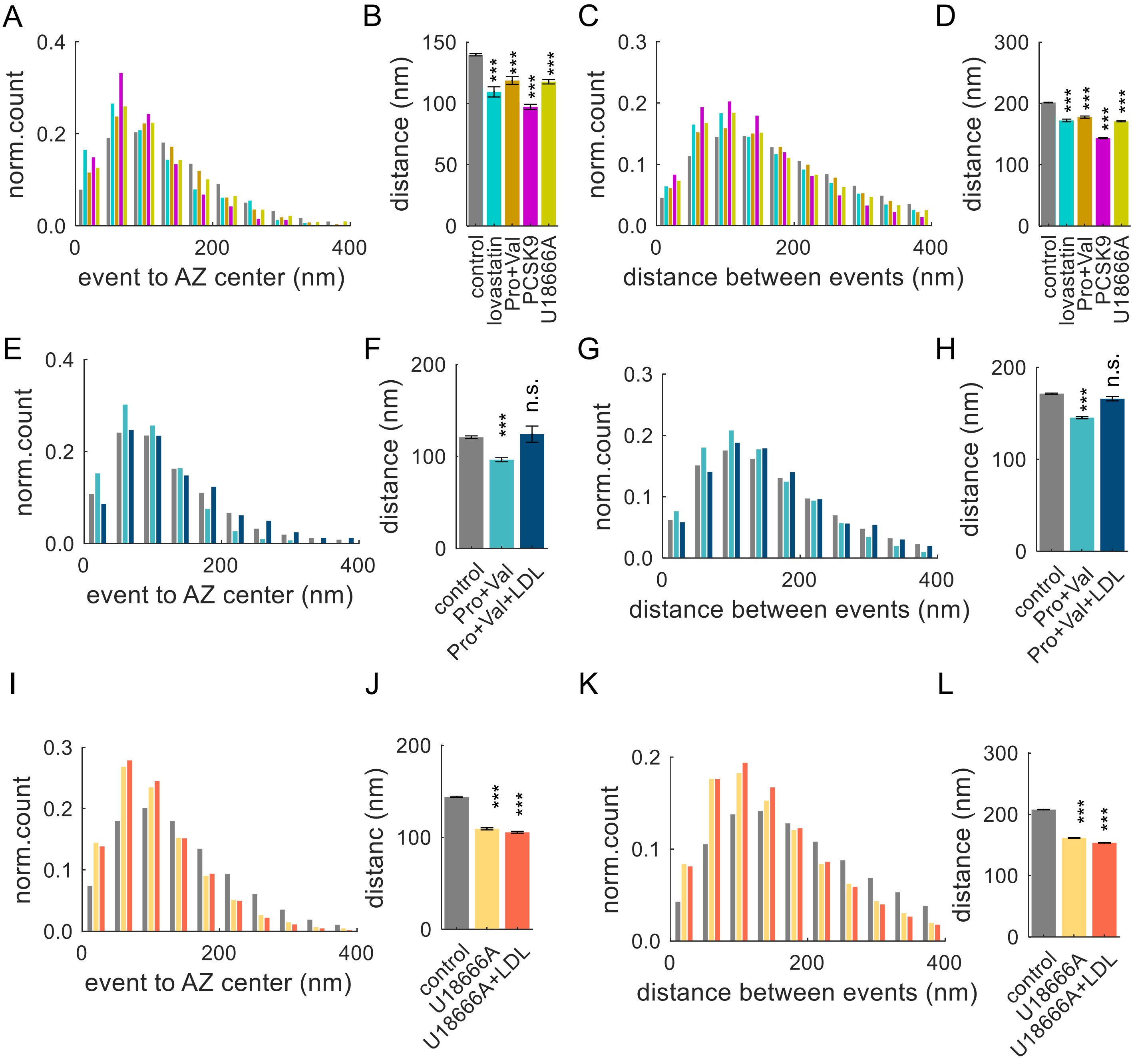

bioRxiv preprint doi: https://doi.org/10.64898/2025.12.28.696787; this version posted February 7, 2026. The copyright holder for this preprint (which was not certified by peer review) is the author/funder, who has granted bioRxiv a license to display the preprint in perpetuity. It is made available under aCC-BY 4.0 International license.

Table S1. Statistical data for figures

P < 0.05 in bold

Figure 1	Condition	mean ± SEM	Sample number	Stastical test	P value
**Figure 1C** ΔF increase	0 mM vs	69.19 ± 1.98	3 (Cultures) / 9 (Coverslips) / 727 (Syn)	One-way ANOVA	**< 0.001**
	0.5 mM (MβCD)	36.94 ± 1.61	3 (Cultures) / 6 (Coverslips) / 440 (Syn)		
	0 mM vs	69.19 ± 1.98	3 (Cultures) / 9 (Coverslips) / 727 (Syn)		**< 0.001**
	2.5 mM (MβCD)	26.36 ± 1.34	3 (Cultures) / 7 (Coverslips) / 373 (Syn)		
	0 mM vs	69.19 ± 1.98	3 (Cultures) / 9 (Coverslips) / 727 (Syn)		**< 0.001**
	10 mM (MβCD)	14.44 ± 1.14	3 (Cultures) / 8 (Coverslips) / 255 (Syn)		
**Figure 1F** ΔF increase	0 mg/ml vs	50.08 ± 1.69	4 (Cultures) / 10 (Coverslips) / 847 (Syn)	One-way ANOVA	**0.044**
	0.1 mg/ml (WSC)	63.08 ± 2.93	4 (Cultures) / 6 (Coverslips) / 562 (Syn)		
	0 mg/ml vs	50.08 ± 1.69	4 (Cultures) / 10 (Coverslips) / 847 (Syn)		**< 0.001**
	0.5 mg/ml (WSC)	69.12 ± 2.54	4 (Cultures) / 11 (Coverslips) / 1326 (Syn)		
	0 mg/ml vs	50.08 ± 1.69	4 (Cultures) / 10 (Coverslips) / 847 (Syn)		**< 0.001**
	1 mg/ml (WSC)	81.66 ± 2.32	4 (Cultures) / 10 (Coverslips) / 1149 (Syn)		
**Figure 1H** ΔF increase	control vs	1.203 ± 0.011	3 (Cultures) / 8 (Coverslips) / 77 (Syn)	One-way ANOVA	**< 0.001**
	MβCD	1.094 ± 0.011	3 (Cultures) / 9 (Coverslips) / 82 (Syn)		
	control vs	1.203 ± 0.011	3 (Cultures) / 8 (Coverslips) / 77 (Syn)		**< 0.001**
	WSC	1.420 ± 0.026	3 (Cultures) / 9 (Coverslips) / 57 (Syn)		
**Figure 1M** UVR	0 mM vs	0.081 ± 0.002	3 (Cultures) / 9 (Coverslips) / 727 (Syn)	One-way ANOVA	0.384
	0.5 mM (MβCD)	0.077 ± 0.002	3 (Cultures) / 6 (Coverslips) / 440 (Syn)		
	0 mM vs	0.081 ± 0.002	3 (Cultures) / 9 (Coverslips) / 727 (Syn)		**0.034**
	2.5 mM (MβCD)	0.074 ± 0.002	3 (Cultures) / 7 (Coverslips) / 373 (Syn)		
	0 mM vs	0.081 ± 0.002	3 (Cultures) / 9 (Coverslips) / 727 (Syn)		**< 0.001**
	10 mM (MβCD)	0.069 ± 0.001	3 (Cultures) / 8 (Coverslips) / 255 (Syn)		
**Figure 1N** MVR Pr	0 mM vs	0.115 ± 0.007	3 (Cultures) / 9 (Coverslips) / 727 (Syn)	One-way ANOVA	**< 0.001**
	0.5 mM (MβCD)	0.022 ± 0.004	3 (Cultures) / 6 (Coverslips) / 440 (Syn)		
	0 mM vs	0.115 ± 0.007	3 (Cultures) / 9 (Coverslips) / 727 (Syn)		**< 0.001**
	2.5 mM (MβCD)	0.006 ± 0.002	3 (Cultures) / 7 (Coverslips) / 373 (Syn)		
	0 mM vs	0.115 ± 0.007	3 (Cultures) / 9 (Coverslips) / 727 (Syn)		**< 0.001**
	10 mM (MβCD)	0.008 ± 0.001	3 (Cultures) / 8 (Coverslips) / 255 (Syn)		
**Figure 1P** UVR	0 mg/ml vs	0.077 ± 0.002	4 (Cultures) / 10 (Coverslips) / 847 (Syn)	One-way ANOVA	0.988
	0.1 mg/ml (WSC)	0.076 ± 0.002	4 (Cultures) / 6 (Coverslips) / 562 (Syn)		
	0 mg/ml vs	0.077 ± 0.002	4 (Cultures) / 10 (Coverslips) / 847 (Syn)		0.166
	0.5 mg/ml (WSC)	0.072 ± 0.002	4 (Cultures) / 11 (Coverslips) / 1326 (Syn)		
	0 mg/ml vs	0.077 ± 0.002	4 (Cultures) / 10 (Coverslips) / 847 (Syn)		0.538
	1 mg/ml (WSC)	0.080 ± 0.002	4 (Cultures) / 10 (Coverslips) / 1149 (Syn)		
**Figure 1Q** MVR Pr	0 mg/ml vs	0.109 ± 0.008	4 (Cultures) / 10 (Coverslips) / 847 (Syn)	One-way ANOVA	**0.0037**
	0.1 mg/ml (WSC)	0.161 ± 0.012	4 (Cultures) / 6 (Coverslips) / 562 (Syn)		
	0 mg/ml vs	0.109 ± 0.008	4 (Cultures) / 10 (Coverslips) / 847 (Syn)		**< 0.001**
	0.5 mg/ml (WSC)	0.174 ± 0.010	4 (Cultures) / 11 (Coverslips) / 1326 (Syn)		
	0 mg/ml vs	0.109 ± 0.008	4 (Cultures) / 10 (Coverslips) / 847 (Syn)		**0.001**
	1 mg/ml (WSC)	0.148 ± 0.010	4 (Cultures) / 10 (Coverslips) / 1149 (Syn)		

Figure 2	Condition	mean ± SEM	Sample number	Stastical test	P value
**Figure 2B** tau (s)	0 mM vs	0.524 ± 0.007	3 (Cultures) / 9 (Coverslips) / 727 (Syn)	One-way ANOVA	**< 0.001**
	0.5 mM (MβCD)	0.436 ± 0.012	3 (Cultures) / 6 (Coverslips) / 440 (Syn)		
	0 mM vs	0.524 ± 0.007	3 (Cultures) / 9 (Coverslips) / 727 (Syn)		**< 0.001**
	2.5 mM (MβCD)	0.402 ± 0.015	3 (Cultures) / 7 (Coverslips) / 373 (Syn)		
	0 mM vs	0.524 ± 0.007	3 (Cultures) / 9 (Coverslips) / 727 (Syn)		**< 0.001**
	10 mM (MβCD)	0.423 ± 0.011	3 (Cultures) / 8 (Coverslips) / 255 (Syn)		
**Figure 2D** ultrafast ratio	0 mM (MβCD)	0.164	3 (Cultures) / 9 (Coverslips) / 727 (Syn)		
	0.5 mM (MβCD)	0.266	3 (Cultures) / 6 (Coverslips) / 440 (Syn)		
	2.5 mM (MβCD)	0.314	3 (Cultures) / 7 (Coverslips) / 373 (Syn)		
	0.5 mM (MβCD)	0.324	3 (Cultures) / 8 (Coverslips) / 255 (Syn)		
**Figure 2F** tau (s)	0 mg/ml vs	0.506 ± 0.009	4 (Cultures) / 10 (Coverslips) / 847 (Syn)	One-way ANOVA	**0.041**
	0.1 mg/ml (WSC)	0.544 ± 0.011	4 (Cultures) / 6 (Coverslips) / 562 (Syn)		
	0 mg/ml vs	0.506 ± 0.009	4 (Cultures) / 10 (Coverslips) / 847 (Syn)		**< 0.001**
	0.5 mg/ml (WSC)	0.552 ± 0.008	4 (Cultures) / 11 (Coverslips) / 1326 (Syn)		
	0 mg/ml vs	0.506 ± 0.009	4 (Cultures) / 10 (Coverslips) / 847 (Syn)		**< 0.001**
	1 mg/ml (WSC)	0.595 ± 0.008	4 (Cultures) / 10 (Coverslips) / 1149 (Syn)		
**Figure 2H** ultrafast ratio	0 mg/ml	0.185	4 (Cultures) / 10 (Coverslips) / 847 (Syn)		
	0.1 mg/ml (WSC)	0.174	4 (Cultures) / 6 (Coverslips) / 562 (Syn)		
	0.5 mg/ml (WSC)	0.162	4 (Cultures) / 11 (Coverslips) / 1326 (Syn)		
	1 mg/ml (WSC)	0.144	4 (Cultures) / 10 (Coverslips) / 1149 (Syn)		
**Figure 2L** fraction of retrieval	0 mM vs	0.954 ± 0.006	3 (Cultures) / 9 (Coverslips) / 727 (Syn)	One-way ANOVA	**< 0.001**
	0.5 mM (MβCD)	1.038 ± 0.012	3 (Cultures) / 6 (Coverslips) / 440 (Syn)		
	0 mM vs	0.954 ± 0.006	3 (Cultures) / 9 (Coverslips) / 727 (Syn)		**< 0.001**
	2.5 mM (MβCD)	1.098 ± 0.016	3 (Cultures) / 7 (Coverslips) / 373 (Syn)		
	0 mM vs	0.954 ± 0.006	3 (Cultures) / 9 (Coverslips) / 727 (Syn)		**< 0.001**
	10 mM (MβCD)	1.143 ± 0.012	3 (Cultures) / 8 (Coverslips) / 255 (Syn)		
**Figure 2N** fraction of retrieval	0 mg/ml vs	0.963 ± 0.009	4 (Cultures) / 10 (Coverslips) / 847 (Syn)	One-way ANOVA	0.967
	0.1 mg/ml (WSC)	0.962 ± 0.011	4 (Cultures) / 6 (Coverslips) / 562 (Syn)		
	0 mg/ml vs	0.963 ± 0.009	4 (Cultures) / 10 (Coverslips) / 847 (Syn)		0.422
	0.5 mg/ml (WSC)	0.950 ± 0.009	4 (Cultures) / 11 (Coverslips) / 1326 (Syn)		
	0 mg/ml vs	0.963 ± 0.009	4 (Cultures) / 10 (Coverslips) / 847 (Syn)		**0.015**
	1 mg/ml (WSC)	0.934 ± 0.008	4 (Cultures) / 10 (Coverslips) / 1149 (Syn)		

Figure 3	Condition	mean ± SEM	Sample number	Stastical test	P value
**Figure 3C** ΔF increase	control vs	39.43 ± 1.25	5 (Cultures) / 11 (Coverslips) / 443 (Syn)	One-way ANOVA	**< 0.001**
	lovastatin	15.22 ± 0.84	5 (Cultures) / 9 (Coverslips) / 338 (Syn)		
	control vs	39.43 ± 1.25	5 (Cultures) / 11 (Coverslips) / 443 (Syn)		**< 0.001**
	Probucol + valspodar	5.54 ± 0.91	5 (Cultures) / 11 (Coverslips) / 349 (Syn)		
	control vs	39.43 ± 1.25	5 (Cultures) / 11 (Coverslips) / 443 (Syn)		**< 0.001**

bioRxiv preprint doi: https://doi.org/10.64898/2025.12.28.696787; this version posted February 7, 2026. The copyright holder for this preprint (which was not certified by peer review) is the author/funder, who has granted bioRxiv a license to display the preprint in perpetuity. It is made available under aCC-BY 4.0 International license.

	PCSK9	6.77 ± 1.53	5 (Cultures) / 9 (Coverslips) / 160 (Syn)		< 0.001
	control vs	39.43 ± 1.25	5 (Cultures) / 11 (Coverslips) / 443 (Syn)		**< 0.001**
	U18666A	15.38 ± 1.19	5 (Cultures) / 7 (Coverslips) / 259 (Syn)		
**Figure 3D** UVR Pr	control vs	0.086 ± 0.002	5 (Cultures) / 11 (Coverslips) / 443 (Syn)	One-way ANOVA	**0.002**
	lovastatin	0.065 ± 0.003	5 (Cultures) / 9 (Coverslips) / 338 (Syn)		
	control vs	0.086 ± 0.002	5 (Cultures) / 11 (Coverslips) / 443 (Syn)		**0.001**
	Probucol + valspodar	0.075 ± 0.005	5 (Cultures) / 11 (Coverslips) / 349 (Syn)		
	control vs	0.086 ± 0.002	5 (Cultures) / 11 (Coverslips) / 443 (Syn)		**0.006**
	PCSK9	0.074 ± 0.005	5 (Cultures) / 9 (Coverslips) / 160 (Syn)		
	control vs	0.086 ± 0.002	5 (Cultures) / 11 (Coverslips) / 443 (Syn)		**0.001**
	U18666A	0.075 ± 0.002	5 (Cultures) / 7 (Coverslips) / 259 (Syn)		
**Figure 4E** MVR Pr	control vs	0.017 ± 0.002	5 (Cultures) / 11 (Coverslips) / 443 (Syn)	One-way ANOVA	**0.005**
	lovastatin	0.001 ± 0.001	5 (Cultures) / 9 (Coverslips) / 338 (Syn)		
	control vs	0.017 ± 0.002	5 (Cultures) / 11 (Coverslips) / 443 (Syn)		**< 0.001**
	Probucol + valspodar	0.001 ± 0.001	5 (Cultures) / 11 (Coverslips) / 349 (Syn)		
	control vs	0.017 ± 0.002	5 (Cultures) / 11 (Coverslips) / 443 (Syn)		**0.039**
	PCSK9	0.009 ± 0.002	5 (Cultures) / 9 (Coverslips) / 160 (Syn)		
	control vs	0.017 ± 0.002	5 (Cultures) / 11 (Coverslips) / 443 (Syn)		**< 0.001**
	U18666A	0.001 ± 0.001	5 (Cultures) / 7 (Coverslips) / 259 (Syn)		
**Figure 3G** ΔF increase	control vs	60.60 ± 1.86	3 (Cultures) / 5 (Coverslips) / 151 (Syn)	One-way ANOVA	**< 0.001**
	pro + val	41.89 ± 4.12	3 (Cultures) / 4 (Coverslips) / 73 (Syn)		
	control vs	60.60 ± 1.86	3 (Cultures) / 5 (Coverslips) / 151 (Syn)		0.25
	pro + val + LDL	57.89 ± 5.68	3 (Cultures) / 4 (Coverslips) / 54 (Syn)		
**Figure 3H** UVR Pr	control vs	0.071 ± 0.002	3 (Cultures) / 5 (Coverslips) / 151 (Syn)	One-way ANOVA	**0.002**
	pro + val	0.061 ± 0.005	3 (Cultures) / 4 (Coverslips) / 73 (Syn)		
	control vs	0.071 ± 0.002	3 (Cultures) / 5 (Coverslips) / 151 (Syn)		**0.002**
	pro + val + LDL	0.072 ± 0.003	3 (Cultures) / 4 (Coverslips) / 54 (Syn)		
**Figure 3I** MVR Pr	control vs	0.101 ± 0.001	3 (Cultures) / 5 (Coverslips) / 151 (Syn)	One-way ANOVA	**< 0.001**
	pro + val	0.021 ± 0.001	3 (Cultures) / 4 (Coverslips) / 73 (Syn)		
	control vs	0.101 ± 0.001	3 (Cultures) / 5 (Coverslips) / 151 (Syn)		0.197
	pro + val + LDL	0.013 ± 0.012	3 (Cultures) / 4 (Coverslips) / 54 (Syn)		
**Figure 3K** ΔF increase	control vs	56.58 ± 2.72	4 (Cultures) / 10 (Coverslips) / 615 (Syn)	One-way ANOVA	**< 0.001**
	U18666A	7.22 ± 2.53	4 (Cultures) / 9 (Coverslips) / 71 (Syn)		
	control vs	56.58 ± 2.72	4 (Cultures) / 10 (Coverslips) / 615 (Syn)		**< 0.001**
	U18666A + LDL	9.88 ± 2.31	4 (Cultures) / 9 (Coverslips) / 181 (Syn)		
**Figure 3L** UVR Pr	control vs	0.077 ± 0.001	4 (Cultures) / 10 (Coverslips) / 362 (Syn)	One-way ANOVA	0.75
	U18666A	0.076 ± 0.002	4 (Cultures) / 9 (Coverslips) / 296 (Syn)		
	control vs	0.077 ± 0.001	4 (Cultures) / 10 (Coverslips) / 362 (Syn)		0.50
	U18666A + LDL	0.074 ± 0.002	4 (Cultures) / 9 (Coverslips) / 250 (Syn)		
**Figure 3M** MVR Pr	control vs	0.014 ± 0.001	4 (Cultures) / 10 (Coverslips) / 362 (Syn)	One-way ANOVA	**< 0.001**
	U18666A	0.005 ± 0.001	4 (Cultures) / 9 (Coverslips) / 296 (Syn)		
	control vs	0.014 ± 0.001	4 (Cultures) / 10 (Coverslips) / 362 (Syn)		**< 0.001**
	U18666A + LDL	0.008 ± 0.001	4 (Cultures) / 9 (Coverslips) / 250 (Syn)		

**Figure 4**	**Condition**	**mean ± SEM**	**Sample number**	**Stastical test**	**P value**
**Figure 4B** tau (s)	control vs	0.542 ± 0.009	5 (Cultures) / 11 (Coverslips) / 443 (Syn)	One-way ANOVA	**< 0.001**
	lovastatin	0.394 ±0.019	5 (Cultures) / 9 (Coverslips) / 338 (Syn)		
	control vs	0.542 ± 0.009	5 (Cultures) / 11 (Coverslips) / 443 (Syn)		**< 0.001**
	Probucol + valspodar	0.444 ± 0.029	5 (Cultures) / 11 (Coverslips) / 349 (Syn)		
	control vs	0.542 ± 0.009	5 (Cultures) / 11 (Coverslips) / 443 (Syn)		**< 0.001**
	PCSK9	0.459 ± 0.024	5 (Cultures) / 9 (Coverslips) / 160 (Syn)		
	control vs	0.542 ± 0.009	5 (Cultures) / 11 (Coverslips) / 443 (Syn)		**< 0.001**
	U18666A	0.444 ± 0.029	5 (Cultures) / 7 (Coverslips) / 259 (Syn)		
**Figure 4D** ultrafast ratio	control	0.176	5 (Cultures) / 11 (Coverslips) / 443 (Syn)		
	lovastatin	0.341	5 (Cultures) / 9 (Coverslips) / 338 (Syn)		
	Probucol + valspodar	0.285	5 (Cultures) / 11 (Coverslips) / 349 (Syn)		
	PCSK9	0.341	5 (Cultures) / 9 (Coverslips) / 160 (Syn)		
	U18666A	0.336	5 (Cultures) / 7 (Coverslips) / 259 (Syn)		
**Figure 4F** tau (s)	control vs	0.528 ± 0.006	4 (Cultures) / 10 (Coverslips) / 615 (Syn)	One-way ANOVA	**< 0.001**
	pro + val	0.422 ± 0.028	4 (Cultures) / 9 (Coverslips) / 71 (Syn)		
	control vs	0.528 ± 0.006	4 (Cultures) / 10 (Coverslips) / 615 (Syn)		0.847
	pro + val + LDL	0.545 ± 0.052	4 (Cultures) / 9 (Coverslips) / 181 (Syn)		
**Figure 4H** ultrafast ratio	control	0.181	4 (Cultures) / 10 (Coverslips) / 615 (Syn)		
	pro + val	0.362	4 (Cultures) / 9 (Coverslips) / 71 (Syn)		
	pro + val + LDL	0.181	4 (Cultures) / 9 (Coverslips) / 181 (Syn)		
**Figure 4J** tau (s)	control vs	0.517 ± 0.008	4 (Cultures) / 10 (Coverslips) / 615 (Syn)	One-way ANOVA	**< 0.001**
	U18666A	0.439 ± 0.016	4 (Cultures) / 9 (Coverslips) / 71 (Syn)		
	control vs	0.517 ± 0.008	4 (Cultures) / 10 (Coverslips) / 615 (Syn)		0.847
	U18666A + LDL	0.440 ± 0.014	4 (Cultures) / 9 (Coverslips) / 181 (Syn)		
**Figure 4L** ultrafast ratio	control	0.185	4 (Cultures) / 10 (Coverslips) / 615 (Syn)		
	U18666A	0.334	4 (Cultures) / 9 (Coverslips) / 71 (Syn)		
	U18666A + LDL	0.284	4 (Cultures) / 9 (Coverslips) / 181 (Syn)		
**Figure 4M** fraction of retreival	control vs	0.954 ± 0.006	5 (Cultures) / 11 (Coverslips) / 443 (Syn)	One-way ANOVA	**< 0.001**
	lovastatin	1.086 ± 0.018	5 (Cultures) / 9 (Coverslips) / 338 (Syn)		
	control vs	0.954 ± 0.006	5 (Cultures) / 11 (Coverslips) / 443 (Syn)		**< 0.001**
	Probucol + valspodar	1.138 ± 0.039	5 (Cultures) / 11 (Coverslips) / 349 (Syn)		
	control vs	0.954 ± 0.006	5 (Cultures) / 11 (Coverslips) / 443 (Syn)		**< 0.001**
	PCSK9	1.132 ± 0.026	5 (Cultures) / 9 (Coverslips) / 160 (Syn)		
	control vs	0.954 ± 0.006	5 (Cultures) / 11 (Coverslips) / 443 (Syn)		**0.028**
	U18666A	1.017 ± 0.020	5 (Cultures) / 7 (Coverslips) / 259 (Syn)		
**Figure 4N** fraction of retreival	control vs	0.958 ± 0.006	3 (Cultures) / 6 (Coverslips) / 429 (Syn)	One-way ANOVA	**< 0.001**
	Pro+Val	1.200 ±0.013	3 (Cultures) / 5 (Coverslips) / 119 (Syn)		
	control vs	0.958 ± 0.006	3 (Cultures) / 6 (Coverslips) / 429 (Syn)		0.847
	Pro+Val+LDL	0.964 ± 0.008	3 (Cultures) / 5 (Coverslips) / 104 (Syn)		
**Figure 4O** fraction of retreival	control vs	0.971 ± 0.007	4 (Cultures) / 10 (Coverslips) / 615 (Syn)	One-way ANOVA	**< 0.001**
	U18666A	1.144 ± 0.016	4 (Cultures) / 9 (Coverslips) / 71 (Syn)		
	control vs	0.971 ± 0.007	4 (Cultures) / 10 (Coverslips) / 615 (Syn)		**< 0.001**

bioRxiv preprint doi: https://doi.org/10.64898/2025.12.28.696787; this version posted February 7, 2026. The copyright holder for this preprint (which was not certified by peer review) is the author/funder, who has granted bioRxiv a license to display the preprint in perpetuity. It is made available under aCC-BY 4.0 International license.

[illegible]	U18666A + LDL	1.135 ± 0.016	4 (Cultures) / 9 (Coverslips) / 181 (Syn)		< 0.001

Figure S1	Condition	mean ± SEM	Sample number	Stastical test	P value
**Figure S1A** Pr	0 mM vs	0.096 ± 0.002	3 (Cultures) / 9 (Coverslips) / 727 (Syn)	One-way ANOVA	**< 0.001**
	0.5 mM (MβCD)	0.078 ± 0.002	3 (Cultures) / 6 (Coverslips) / 440 (Syn)		
	0 mM vs	0.096 ± 0.002	3 (Cultures) / 9 (Coverslips) / 727 (Syn)		**< 0.001**
	2.5 mM (MβCD)	0.075 ± 0.002	3 (Cultures) / 7 (Coverslips) / 373 (Syn)		
	0 mM vs	0.096 ± 0.002	3 (Cultures) / 9 (Coverslips) / 727 (Syn)		**< 0.001**
	10 mM (MβCD)	0.070 ± 0.001	3 (Cultures) / 8 (Coverslips) / 255 (Syn)		
**Figure S1B** Pr	0 mg/ml vs	0.090 ± 0.002	4 (Cultures) / 10 (Coverslips) / 847 (Syn)	One-way ANOVA	0.464
	0.1 mg/ml (WSC)	0.096 ± 0.003	4 (Cultures) / 6 (Coverslips) / 562 (Syn)		
	0 mg/ml vs	0.090 ± 0.002	4 (Cultures) / 10 (Coverslips) / 847 (Syn)		0.744
	0.5 mg/ml (WSC)	0.094 ± 0.003	4 (Cultures) / 11 (Coverslips) / 1326 (Syn)		
	0 mg/ml vs	0.090 ± 0.002	4 (Cultures) / 10 (Coverslips) / 847 (Syn)		**0.031**
	1 mg/ml (WSC)	0.100 ± 0.003	4 (Cultures) / 10 (Coverslips) / 1149 (Syn)		
**Figure S1C** MVR/UVR ratio	0 mM vs	0.206 ± 0.029	3 (Cultures) / 9 (Coverslips) / 727 (Syn)	One-way ANOVA	**< 0.001**
	0.5 mM (MβCD)	0.027 ± 0.006	3 (Cultures) / 6 (Coverslips) / 440 (Syn)		
	0 mM vs	0.206 ± 0.029	3 (Cultures) / 9 (Coverslips) / 727 (Syn)		**< 0.001**
	2.5 mM (MβCD)	0.001 ± 0.001	3 (Cultures) / 7 (Coverslips) / 373 (Syn)		
	0 mM vs	0.206 ± 0.029	3 (Cultures) / 9 (Coverslips) / 727 (Syn)		**< 0.001**
	10 mM (MβCD)	0.001 ± 0.001	3 (Cultures) / 8 (Coverslips) / 255 (Syn)		
**Figure S1D** MVR/UVR ratio	0 mg/ml vs	0.182 ± 0.020	4 (Cultures) / 10 (Coverslips) / 847 (Syn)	One-way ANOVA	**< 0.001**
	0.1 mg/ml (WSC)	0.310 ± 0.030	4 (Cultures) / 6 (Coverslips) / 562 (Syn)		
	0 mg/ml vs	0.182 ± 0.020	4 (Cultures) / 10 (Coverslips) / 847 (Syn)		**< 0.001**
	0.5 mg/ml (WSC)	0.301 ± 0.026	4 (Cultures) / 11 (Coverslips) / 1326 (Syn)		
	0 mg/ml vs	0.182 ± 0.020	4 (Cultures) / 10 (Coverslips) / 847 (Syn)		**< 0.001**
	1 mg/ml (WSC)	0.285 ± 0.052	4 (Cultures) / 10 (Coverslips) / 1149 (Syn)		
**Figure S1E** asynchro	0 mM vs	0.016 ± 0.001	3 (Cultures) / 9 (Coverslips) / 727 (Syn)	One-way ANOVA	0.165
	0.5 mM (MβCD)	0.014 ± 0.001	3 (Cultures) / 6 (Coverslips) / 440 (Syn)		
	0 mM vs	0.016 ± 0.001	3 (Cultures) / 9 (Coverslips) / 727 (Syn)		**< 0.001**
	2.5 mM (MβCD)	0.011 ± 0.001	3 (Cultures) / 7 (Coverslips) / 373 (Syn)		
	0 mM vs	0.016 ± 0.001	3 (Cultures) / 9 (Coverslips) / 727 (Syn)		**< 0.001**
	10 mM (MβCD)	0.011 ± 0.001	3 (Cultures) / 8 (Coverslips) / 255 (Syn)		
**Figure S1F** asynchro	0 mg/ml vs	0.015 ± 0.001	4 (Cultures) / 10 (Coverslips) / 847 (Syn)	One-way ANOVA	0.289
	0.1 mg/ml (WSC)	0.017 ± 0.002	4 (Cultures) / 6 (Coverslips) / 562 (Syn)		
	0 mg/ml vs	0.015 ± 0.001	4 (Cultures) / 10 (Coverslips) / 847 (Syn)		**0.002**
	0.5 mg/ml (WSC)	0.020 ± 0.001	4 (Cultures) / 11 (Coverslips) / 1326 (Syn)		
	0 mg/ml vs	0.015 ± 0.001	4 (Cultures) / 10 (Coverslips) / 847 (Syn)		**0.034**
	1 mg/ml (WSC)	0.018 ± 0.001	4 (Cultures) / 10 (Coverslips) / 1149 (Syn)		

Figure S2	Condition	mean ± SEM	Sample number	Stastical test	P value
**Figure S2B** distance (nm) center to event	0 mM vs	136.1 ± 0.9	3 (Cultures) / 9 (Coverslips) / 727 (Syn)	One-way ANOVA	0.164
	0.5 mM (MβCD)	132.6 ± 1.5	3 (Cultures) / 6 (Coverslips) / 440 (Syn)		
	0 mM vs	136.1 ± 0.9	3 (Cultures) / 9 (Coverslips) / 727 (Syn)		**< 0.001**
	2.5 mM (MβCD)	130.9 ± 1.1	3 (Cultures) / 7 (Coverslips) / 373 (Syn)		
	0 mM vs	136.1 ± 0.9	3 (Cultures) / 9 (Coverslips) / 727 (Syn)		**< 0.001**
	10 mM (MβCD)	116.7 ± 1.3	3 (Cultures) / 8 (Coverslips) / 255 (Syn)		
**Figure S2D** distance (nm) event to event	0 mM vs	197.0 ± 0.4	3 (Cultures) / 9 (Coverslips) / 727 (Syn)	One-way ANOVA	0.991
	0.5 mM (MβCD)	196.7 ± 0.8	3 (Cultures) / 6 (Coverslips) / 440 (Syn)		
	0 mM vs	197.0 ± 0.4	3 (Cultures) / 9 (Coverslips) / 727 (Syn)		0.941
	2.5 mM (MβCD)	196.6 ± 0.6	3 (Cultures) / 7 (Coverslips) / 373 (Syn)		
	0 mM vs	197.0 ± 0.4	3 (Cultures) / 9 (Coverslips) / 727 (Syn)		**< 0.001**
	10 mM (MβCD)	174.8 ± 0.6	3 (Cultures) / 8 (Coverslips) / 255 (Syn)		
**Figure S2F** distance (nm) center to event	0 mg/ml vs	139.4 ± 1.2	4 (Cultures) / 10 (Coverslips) / 847 (Syn)	One-way ANOVA	0.615
	0.1 mg/ml (WSC)	137.1 ± 1.4	4 (Cultures) / 6 (Coverslips) / 562 (Syn)		
	0 mg/ml vs	139.4 ± 1.2	4 (Cultures) / 10 (Coverslips) / 847 (Syn)		0.631
	0.5 mg/ml (WSC)	137.5 ± 1.0	4 (Cultures) / 11 (Coverslips) / 1326 (Syn)		
	0 mg/ml vs	139.4 ± 1.2	4 (Cultures) / 10 (Coverslips) / 847 (Syn)		**< 0.001**
	1 mg/ml (WSC)	149.5 ± 1.1	4 (Cultures) / 10 (Coverslips) / 1149 (Syn)		
**Figure S2H** distance (nm) event to event	0 mg/ml vs	202.6 ± 0.6	4 (Cultures) / 10 (Coverslips) / 847 (Syn)	One-way ANOVA	0.071
	0.1 mg/ml (WSC)	198.6 ± 0.6	4 (Cultures) / 6 (Coverslips) / 562 (Syn)		
	0 mg/ml vs	202.6 ± 0.6	4 (Cultures) / 10 (Coverslips) / 847 (Syn)		0.093
	0.5 mg/ml (WSC)	199.6 ± 0.5	4 (Cultures) / 11 (Coverslips) / 1326 (Syn)		
	0 mg/ml vs	202.6 ± 0.6	4 (Cultures) / 10 (Coverslips) / 847 (Syn)		**< 0.001**
	1 mg/ml (WSC)	214.6 ± 0.5	4 (Cultures) / 10 (Coverslips) / 1149 (Syn)		
**Figure S2I** # release site	0 mM vs	11.318 ± 0.179	3 (Cultures) / 9 (Coverslips) / 727 (Syn)	One-way ANOVA	**< 0.001**
	0.5 mM (MβCD)	10.204 ± 0.219	3 (Cultures) / 6 (Coverslips) / 440 (Syn)		
	0 mM vs	11.318 ± 0.179	3 (Cultures) / 9 (Coverslips) / 727 (Syn)		**< 0.001**
	2.5 mM (MβCD)	9.710 ± 0.117	3 (Cultures) / 7 (Coverslips) / 373 (Syn)		
	0 mM vs	11.318 ± 0.179	3 (Cultures) / 9 (Coverslips) / 727 (Syn)		**< 0.001**
	10 mM (MβCD)	9.745 ± 0.223	3 (Cultures) / 8 (Coverslips) / 255 (Syn)		
**Figure S2J** # event in a release site	0 mM vs	1.477 ± 0.009	3 (Cultures) / 9 (Coverslips) / 727 (Syn)	One-way ANOVA	0.768
	0.5 mM (MβCD)	1.493 ± 0.014	3 (Cultures) / 6 (Coverslips) / 440 (Syn)		
	0 mM vs	1.477 ± 0.009	3 (Cultures) / 9 (Coverslips) / 727 (Syn)		**< 0.001**
	2.5 mM (MβCD)	1.393 ± 0.010	3 (Cultures) / 7 (Coverslips) / 373 (Syn)		
	0 mM vs	1.477 ± 0.009	3 (Cultures) / 9 (Coverslips) / 727 (Syn)		**< 0.001**
	10 mM (MβCD)	1.406 ± 0.015	3 (Cultures) / 8 (Coverslips) / 255 (Syn)		
**Figure S2K** # release site	0 mg/ml vs	11.300 ± 0.219	4 (Cultures) / 10 (Coverslips) / 847 (Syn)	One-way ANOVA	0.226
	0.1 mg/ml (WSC)	12.039 ± 0.305	4 (Cultures) / 6 (Coverslips) / 562 (Syn)		
	0 mg/ml vs	11.300 ± 0.219	4 (Cultures) / 10 (Coverslips) / 847 (Syn)		0.922
	0.5 mg/ml (WSC)	11.513 ± 0.222	4 (Cultures) / 11 (Coverslips) / 1326 (Syn)		
	0 mg/ml vs	11.300 ± 0.219	4 (Cultures) / 10 (Coverslips) / 847 (Syn)		**0.002**
	1 mg/ml (WSC)	12.499 ± 0.268	4 (Cultures) / 10 (Coverslips) / 1149 (Syn)		
**Figure S2L** # event in a	0 mg/ml vs	1.432 ± 0.012	4 (Cultures) / 10 (Coverslips) / 847 (Syn)	One-way ANOVA	0.828
	0.1 mg/ml (WSC)	1.447 ± 0.015	4 (Cultures) / 6 (Coverslips) / 562 (Syn)		
	0 mg/ml vs	1.432 ± 0.012	4 (Cultures) / 10 (Coverslips) / 847 (Syn)		**0.009**

bioRxiv preprint doi: https://doi.org/10.64898/2025.12.28.696787; this version posted February 7, 2026. The copyright holder for this preprint (which was not certified by peer review) is the author/funder, who has granted bioRxiv a license to display the preprint in perpetuity. It is made available under aCC-BY 4.0 International license.

# event in a release site	0.5 mg/ml (WSC)	1.492 ± 0.012	4 (Cultures) / 11 (Coverslips) / 1326 (Syn)	[illegible]	[illegible]
	0 mg/ml vs	1.432 ± 0.012	4 (Cultures) / 10 (Coverslips) / 847 (Syn)		**0.003**
	1 mg/ml (WSC)	1.490 ± 0.011	4 (Cultures) / 10 (Coverslips) / 1149 (Syn)		

Figure S3	Condition	mean ± SEM	Sample number	Stastical test	P value
**Figure S3C** dwell time (s)	0 mM vs	0.125 ± 0.003	3 (Cultures) / 9 (Coverslips) / 727 (Syn)	One-way ANOVA	**< 0.001**
	0.5 mM (MβCD)	0.088 ± 0.005	3 (Cultures) / 6 (Coverslips) / 440 (Syn)		
	0 mM vs	0.125 ± 0.003	3 (Cultures) / 9 (Coverslips) / 727 (Syn)		**< 0.001**
	2.5 mM (MβCD)	0.075 ± 0.007	3 (Cultures) / 7 (Coverslips) / 373 (Syn)		
	0 mM vs	0.125 ± 0.003	3 (Cultures) / 9 (Coverslips) / 727 (Syn)		**< 0.001**
	10 mM (MβCD)	0.080 ± 0.005	3 (Cultures) / 8 (Coverslips) / 255 (Syn)		
**Figure S3D** half time (s)	0 mM vs	0.417 ± 0.005	3 (Cultures) / 9 (Coverslips) / 727 (Syn)	One-way ANOVA	**< 0.001**
	0.5 mM (MβCD)	0.349 ± 0.010	3 (Cultures) / 6 (Coverslips) / 440 (Syn)		
	0 mM vs	0.417 ± 0.005	3 (Cultures) / 9 (Coverslips) / 727 (Syn)		**< 0.001**
	2.5 mM (MβCD)	0.301 ± 0.013	3 (Cultures) / 7 (Coverslips) / 373 (Syn)		
	0 mM vs	0.417 ± 0.005	3 (Cultures) / 9 (Coverslips) / 727 (Syn)		**< 0.001**
	10 mM (MβCD)	0.316 ± 0.009	3 (Cultures) / 8 (Coverslips) / 255 (Syn)		
**Figure S3E** dwell time (s)	0 mg/ml vs	0.124 ± 0.004	4 (Cultures) / 10 (Coverslips) / 847 (Syn)	One-way ANOVA	**0.1377**
	0.1 mg/ml (WSC)	0.136 ± 0.005	4 (Cultures) / 6 (Coverslips) / 562 (Syn)		
	0 mg/ml vs	0.124 ± 0.004	4 (Cultures) / 10 (Coverslips) / 847 (Syn)		0.9362
	0.5 mg/ml (WSC)	0.124 ± 0.004	4 (Cultures) / 11 (Coverslips) / 1326 (Syn)		
	0 mg/ml vs	0.124 ± 0.004	4 (Cultures) / 10 (Coverslips) / 847 (Syn)		**0.02121**
	1 mg/ml (WSC)	0.146 ± 0.004	4 (Cultures) / 10 (Coverslips) / 1149 (Syn)		
**Figure S3F** half time (s)	0 mg/ml vs	0.408 ± 0.008	4 (Cultures) / 10 (Coverslips) / 847 (Syn)	One-way ANOVA	0.9424
	0.1 mg/ml (WSC)	0.424 ± 0.009	4 (Cultures) / 6 (Coverslips) / 562 (Syn)		
	0 mg/ml vs	0.408 ± 0.008	4 (Cultures) / 10 (Coverslips) / 847 (Syn)		0.5955
	0.5 mg/ml (WSC)	0.405 ± 0.007	4 (Cultures) / 11 (Coverslips) / 1326 (Syn)		
	0 mg/ml vs	0.408 ± 0.008	4 (Cultures) / 10 (Coverslips) / 847 (Syn)		**0.0315**
	1 mg/ml (WSC)	0.435 ± 0.007	4 (Cultures) / 10 (Coverslips) / 1149 (Syn)		

Figure S5	Condition	mean ± SEM	Sample number	Stastical test	P value
**Figure S5A** Pr	control vs	0.098 ± 0.003	5 (Cultures) / 11 (Coverslips) / 443 (Syn)	One-way ANOVA	**< 0.001**
	lovastatin	0.065 ± 0.003	5 (Cultures) / 9 (Coverslips) / 338 (Syn)		
	control vs	0.098 ± 0.003	5 (Cultures) / 11 (Coverslips) / 443 (Syn)		**< 0.001**
	probucol + valspodar	0.067 ± 0.003	5 (Cultures) / 9 (Coverslips) / 160 (Syn)		
	control vs	0.098 ± 0.003	5 (Cultures) / 11 (Coverslips) / 443 (Syn)		**0.041**
	PCSK9	0.086 ± 0.003	5 (Cultures) / 7 (Coverslips) / 259 (Syn)		
	control vs	0.098 ± 0.003	5 (Cultures) / 11 (Coverslips) / 443 (Syn)		**0.0014**
	U18666A	0.081 ± 0.002	5 (Cultures) / 5 (Coverslips) / 160 (Syn)		
**Figure S5B** MVR/UVR ratio	control vs	0.186 ± 0.015	5 (Cultures) / 11 (Coverslips) / 443 (Syn)	One-way ANOVA	**< 0.001**
	lovastatin	0.003 ± 0.003	5 (Cultures) / 9 (Coverslips) / 338 (Syn)		
	control vs	0.186 ± 0.015	5 (Cultures) / 11 (Coverslips) / 443 (Syn)		**< 0.001**
	probucol + valspodar	0.010 ± 0.006	5 (Cultures) / 9 (Coverslips) / 160 (Syn)		
	control vs	0.186 ± 0.015	5 (Cultures) / 11 (Coverslips) / 443 (Syn)		**0.039**
	PCSK9	0.093 ± 0.020	5 (Cultures) / 7 (Coverslips) / 259 (Syn)		
	control vs	0.186 ± 0.015	5 (Cultures) / 11 (Coverslips) / 443 (Syn)		**< 0.001**
	U18666A	0.001 ± 0.001	5 (Cultures) / 5 (Coverslips) / 160 (Syn)		
**Figure S5C** Pr	control vs	0.088 ± 0.003	5 (Cultures) / 10 (Coverslips) / 893 (Syn)	Two-sample t-test	0.291
	neuron-only	0.075 ± 0.004	5 (Cultures) / 10 (Coverslips) / 50 (Syn)		
**Figure S5D** UVR Pr	control vs	0.070 ± 0.071	5 (Cultures) / 10 (Coverslips) / 893 (Syn)	Two-sample t-test	0.631
	neuron-only	0.072 ± 0.003	5 (Cultures) / 10 (Coverslips) / 50 (Syn)		
**Figure S5E** MVR Pr	control vs	0.018 ± 0.002	5 (Cultures) / 10 (Coverslips) / 893 (Syn)	Two-sample t-test	**< 0.001**
	neuron-only	0.004 ± 0.001	5 (Cultures) / 10 (Coverslips) / 50 (Syn)		
**Figure S5F** MVR/UVR ratio	control vs	0.153 ± 0.013	3 (Cultures) / 5 (Coverslips) / 151 (Syn)	One-way ANOVA	**0.048**
	Pro+Val	0.013 ± 0.001	3 (Cultures) / 4 (Coverslips) / 73 (Syn)		
	control vs	0.153 ± 0.013	3 (Cultures) / 5 (Coverslips) / 151 (Syn)		0.2171
	Pro+Val+LDL	0.200 ± 0.027	3 (Cultures) / 4 (Coverslips) / 54 (Syn)		
**Figure S5G** MVR/UVR ratio	control vs	0.232 ± 0.027	4 (Cultures) / 10 (Coverslips) / 615 (Syn)	One-way ANOVA	**< 0.001**
	U18666A	0.090 ± 0.025	4 (Cultures) / 9 (Coverslips) / 71 (Syn)		
	control vs	0.232 ± 0.027	4 (Cultures) / 10 (Coverslips) / 615 (Syn)		**< 0.001**
	U18666AI+LDL	0.109 ± 0.014	4 (Cultures) / 9 (Coverslips) / 181 (Syn)		

Figure S6	Condition	mean ± SEM	Sample number	Stastical test	P value
**Figure S6B** distance (nm) center to event	control vs	139.6 ± 0.9	5 (Cultures) / 11 (Coverslips) / 443 (Syn)	One-way ANOVA	**< 0.001**
	lovastatin	109.3 ± 4.2	5 (Cultures) / 9 (Coverslips) / 338 (Syn)		
	control vs	139.6 ± 0.9	5 (Cultures) / 11 (Coverslips) / 443 (Syn)		**< 0.001**
	Pro+Val	118.6 ± 3.2	5 (Cultures) / 9 (Coverslips) / 160 (Syn)		
	control vs	139.6 ± 0.9	5 (Cultures) / 11 (Coverslips) / 443 (Syn)		**< 0.001**
	PCSK9	97.1 ± 2.1	5 (Cultures) / 7 (Coverslips) / 259 (Syn)		
	control vs	139.6 ± 0.9	5 (Cultures) / 11 (Coverslips) / 443 (Syn)		**< 0.001**
	U18666A	117.6 ± 1.8	5 (Cultures) / 5 (Coverslips) / 160 (Syn)		
**Figure S6D** distance (nm) center to event	control vs	201.3 ± 0.4	5 (Cultures) / 11 (Coverslips) / 443 (Syn)	One-way ANOVA	**< 0.001**
	lovastatin	172.1 ± 2.2	5 (Cultures) / 9 (Coverslips) / 338 (Syn)		
	control vs	201.3 ± 0.4	5 (Cultures) / 11 (Coverslips) / 443 (Syn)		**< 0.001**
	Pro+Val	177.6 ± 1.8	5 (Cultures) / 9 (Coverslips) / 160 (Syn)		
	control vs	201.3 ± 0.4	5 (Cultures) / 11 (Coverslips) / 443 (Syn)		**< 0.001**
	PCSK9	143.3 ± 1.0	5 (Cultures) / 7 (Coverslips) / 259 (Syn)		
	control vs	201.3 ± 0.4	5 (Cultures) / 11 (Coverslips) / 443 (Syn)		**< 0.001**
	U18666A	170.7 ± 0.9	5 (Cultures) / 5 (Coverslips) / 160 (Syn)		
**Figure S6F** distance (nm) center to event	control vs	120.8 ± 1.4	3 (Cultures) / 6 (Coverslips) / 429 (Syn)	One-way ANOVA	**< 0.001**
	Pro+Val	96.3 ± 2.2	3 (Cultures) / 5 (Coverslips) / 119 (Syn)		
	control vs	120.8 ± 1.4	3 (Cultures) / 6 (Coverslips) / 429 (Syn)		0.9151
	Pro+Val+LDL	124.1 ± 8.8	3 (Cultures) / 5 (Coverslips) / 104 (Syn)		
**Figure S6H** distance (nm) center to event	control vs	171.5 ± 0.6	3 (Cultures) / 6 (Coverslips) / 429 (Syn)	One-way ANOVA	**< 0.001**
	Pro+Val	145.3 ± 1.2	3 (Cultures) / 5 (Coverslips) / 119 (Syn)		
	control vs	171.5 ± 0.6	3 (Cultures) / 6 (Coverslips) / 429 (Syn)		0.0857
	[illegible]				

bioRxiv preprint doi: https://doi.org/10.64898/2025.12.28.696787; this version posted February 7, 2026. The copyright holder for this preprint (which was not certified by peer review) is the author/funder, who has granted bioRxiv a license to display the preprint in perpetuity. It is made available under aCC-BY 4.0 International license.

[illegible]	Pro+Val+LDL	165.9 ± 2.4	3 (Cultures) / 5 (Coverslips) / 104 (Syn)		[illegible]
**Figure S6J** distance (nm) center to event	control vs U18666A	144.0 ± 0.7	4 (Cultures) / 10 (Coverslips) / 615 (Syn)	One-way ANOVA	**< 0.001**
		109.2 ± 1.2	4 (Cultures) / 9 (Coverslips) / 71 (Syn)		
	control vs U18666A+LDL	144.0 ± 0.7	4 (Cultures) / 10 (Coverslips) / 615 (Syn)		**< 0.001**
		105.5 ± 1.0	4 (Cultures) / 9 (Coverslips) / 181 (Syn)		
**Figure S6L** distance (nm) center to event	control vs U18666A	207.7 ± 0.3	4 (Cultures) / 10 (Coverslips) / 615 (Syn)	One-way ANOVA	**< 0.001**
		161.4 ± 0.6	4 (Cultures) / 9 (Coverslips) / 71 (Syn)		
	control vs U18666A+LDL	207.7 ± 0.3	4 (Cultures) / 10 (Coverslips) / 615 (Syn)		**< 0.001**
		153.5 ± 0.4	4 (Cultures) / 9 (Coverslips) / 181 (Syn)		
